# Lessons Learned From the Implementation of a Pilot Study on Self-collected Specimen Return by Sexual Minority Men (Project Caboodle!): Qualitative Exploration

**DOI:** 10.2196/43539

**Published:** 2023-04-06

**Authors:** Gregory Sallabank, Rob Stephenson, Monica Gandhi, Leland Merrill, Akshay Sharma

**Affiliations:** 1 Center for Sexuality and Health Disparities University of Michigan Ann Arbor, MI United States; 2 Department of Systems, Populations and Leadership University of Michigan School of Nursing Ann Arbor, MI United States; 3 Division of HIV, Infectious Disease, and Global Medicine Department of Medicine University of California, San Francisco San Francisco, CA United States; 4 Department of Health Behavior and Biological Sciences University of Michigan School of Nursing Ann Arbor, MI United States

**Keywords:** HIV, sexually transmitted diseases, preexposure prophylaxis, self-testing, sexual and gender minorities

## Abstract

**Background:**

Self-collection of specimens at home and their return by mail might help reduce some of the barriers to HIV and bacterial sexually transmitted infection (STI) screening encountered by gay, bisexual, and other men who have sex with men (GBMSM). To evaluate the benefits and challenges of bringing this approach to scale, researchers are increasingly requesting GBMSM to return self-collected specimens as part of web-based sexual health studies. Testing self-collected hair samples for preexposure prophylaxis drug levels may also be a viable option to identify GBMSM who face adherence difficulties and offer them support.

**Objective:**

Project Caboodle! sought to evaluate the acceptability and feasibility of self-collecting at home and returning by mail 5 specimens (a finger-stick blood sample, a pharyngeal swab, a rectal swab, a urine specimen, and a hair sample) among 100 sexually active GBMSM in the United States aged between 18 and 34 years. In this manuscript, we aimed to describe the key lessons learned from our study’s implementation and to present recommendations offered by participants to maximize the rates of self-collected specimen return.

**Methods:**

Following the specimen self-collection phase, a subset of 25 participants (11 who returned all 5 specimens, 4 who returned between 1 and 4 specimens, and 10 who did not return any specimens) was selected for in-depth interviews conducted via a videoconferencing platform. During the session, a semistructured interview guide was used to discuss the factors influencing decisions regarding returning self-collected specimens for laboratory processing. The transcripts were analyzed using template analysis.

**Results:**

University branding of web-based and physical materials instilled a sense of trust in participants and increased their confidence in the test results. Shipping the specimen self-collection box in plain unmarked packaging promoted discretion during transit and on its receipt. Using different colored bags with matching color-coded instructions to self-collect each type of specimen minimized the potential for confusion. Participants recommended including prerecorded instructional videos to supplement the written instructions, providing information on the importance of triple-site bacterial STI testing, and adding a reminder of the types of testing that would and would not be conducted on hair samples. Participants also suggested tailoring the specimen self-collection box to include only the tests that they might be interested in completing at that time, adding real-time videoconferencing to the beginning of the study to introduce the research team, and sending personalized reminders following the delivery of the specimen self-collection box.

**Conclusions:**

Our results offer valuable insights into aspects that facilitated participant engagement in self-collected specimen return, as well as areas for potential improvement to maximize return rates. Our findings can help guide the design of future large-scale studies and public health programs for home-based HIV, bacterial STI, and preexposure prophylaxis adherence testing.

**International Registered Report Identifier (IRRID):**

RR2-10.2196/13647

## Introduction

### Background

Gay, bisexual, and other men who have sex with men (GBMSM) in the United States are heavily affected by HIV and bacterial sexually transmitted infections (STIs) [1,2]. Sexual network characteristics and behavioral or biological factors (eg, multiple partners, condomless sex, and substance use) are known to increase susceptibility [3-6]. Timely detection and treatment are essential to reducing the burden of these infectious diseases among GBMSM. National recommendations state that all sexually active GBMSM should be screened for HIV, gonorrhea, and chlamydia at least annually and more frequently if warranted (eg, biannually or quarterly) depending on individual risk profiles [7,8]. Triple-site testing for gonorrhea and chlamydia, that is, testing at the pharyngeal, rectal, and urethral sites, is a crucial component of surveillance because STIs in the throat and rectum are often asymptomatic [9-11] and can be missed if urine-only screening is performed [12,13]. Despite concerted efforts by public health agencies at the national, state, and local levels, the annual rates of testing for HIV and bacterial STIs among GBMSM remain suboptimal [14,15].

Individual factors (eg, fear of a positive test result and low-risk perception), structural factors (eg, lack of transportation and limited access to culturally competent health care), socioeconomic factors (eg, stigma, discrimination, and inadequate health insurance coverage), and privacy factors (eg, concerns about being seen by friends or family members at a testing location) are well-documented barriers to HIV and bacterial STI screening among GBMSM [16-18]. Stay-at-home mandates during the early stages of the COVID-19 pandemic posed obstacles to seeking in-person services and resulted in substantially decreased rates of HIV and bacterial STI testing [19], a trend that persisted even after the relaxation of mandates [20]. As nonemergent health care operations return to full capacity, the importance of supplemental approaches to identify new infections among GBMSM and link them to medical care remains high. Self-collection of specimens at home for rapid testing or their return by mail for laboratory processing may help reduce some impediments to HIV and bacterial STI screening [21-23]. Self-collected finger-stick blood samples [24], pharyngeal swabs [25], rectal swabs [26], and urine specimens [27] have been found to be equally valid and reliable as clinician-collected samples for HIV and bacterial STI screening. To evaluate the benefits and challenges of bringing this approach to scale, researchers are increasingly requesting GBMSM to return self-collected specimens as part of web-based sexual health studies [28-32].

Since the 2012 approval of oral preexposure prophylaxis (PrEP) for HIV in the United States, the awareness and use of this prevention tool has steadily increased among GBMSM [33]. PrEP efficacy is highly dependent on adherence to the prescribed regimen [34-36] but taking a daily pill can be onerous for some individuals. Multiple studies conducted among GBMSM have found blood PrEP drug levels corresponding to <4 doses per week after 6 months of PrEP initiation [37-40]. Forgetting to take the medication every day, experiencing side effects (eg, headache and nausea), and missing follow-up appointments have been identified as common reasons for poor adherence [41,42]. In addition to blood tests, some objective measures of PrEP adherence used in previous research include pill counts, pharmacy refills, and electronic adherence monitors [43,44]. One specimen that has recently demonstrated utility in PrEP drug–level testing is hair [45-49]. Segmental analysis of hair samples can allow for an objective assessment of PrEP adherence over different time intervals [50,51]. Given that hair is a nonbiohazardous, easy-to-ship specimen that remains stable at ambient temperature, its self-collection and return for PrEP drug–level testing may be a viable option to identify GBMSM who face adherence difficulties and offer them support.

### Objectives

Project Caboodle! sought to evaluate the acceptability and feasibility of self-collecting at home and returning by mail 5 specimens for HIV, bacterial STI, and potential PrEP drug–level testing among 100 sexually active GBMSM in the United States aged between 18 and 34 years. Complete details pertaining to the study protocol [52] and a description of the study results [53] have been published elsewhere. Participant-related activities were completed between March 2019 and April 2020. In this manuscript, we aimed to describe the key lessons learned from our study’s implementation and to present recommendations offered by participants to maximize the rates of self-collected specimen return based on in-depth interviews with those who chose to return all, some, or none of the specimens.

## Methods

### Participant Recruitment

Participants were recruited via social media advertising on Facebook and Grindr. Individuals who clicked on the advertisements were directed to the study’s landing page that included a brief overview of the protocol and a link to the informed consent form. Those who consented were asked to complete an eligibility screener. The eligibility criteria included being assigned male sex at birth, reporting a male gender identity, being 18 to 34 years of age, residing in the United States or dependent areas, not known to be living with HIV, having ≥2 male sex partners in the past 3 months, and expressing willingness to receive a specimen self-collection box at home. Those who were eligible were asked to provide their contact information (name, email address, mobile phone number, and preferred mailing address). Those who did not provide informed consent, did not meet the eligibility criteria, or did not provide verifiable contact information were directed to the Centers for Disease Control and Prevention website containing information and resources on HIV and bacterial STIs.

### Study Procedures

In the first phase, 100 participants who completed a web-based survey were shipped a box containing instructions and materials to self-collect and return any of the following: a finger-stick blood sample (for HIV testing), a pharyngeal swab, a rectal swab, a urine specimen (for triple-site gonorrhea and chlamydia testing), and a hair sample (to assess its adequacy for potential PrEP drug–level testing). Participants were given a period of 6 weeks from receiving the box to returning self-collected specimens of their choice for laboratory processing by using envelopes affixed with prepaid FedEx shipping labels. No incentive, monetary or otherwise, was provided for completing this step. Test results were delivered back to participants by a counselor via a phone call or an email containing a link to a secure Box folder created specifically for each participant.

In the second phase, a subset of 25% (25/100) of participants (those who returned all 5 specimens: 11/39, 28%; those who returned between 1 and 4 specimens: 4 /12, 33%; and those who did not return any specimens: 10/49, 20%) was selected for in-depth interviews conducted via BlueJeans (Verizon Communications), a videoconferencing platform that allows compliance with the Health Insurance Portability and Accountability Act. Purposive sampling was used to ensure variations in age, race, and ethnicity. During the session, a semistructured interview guide was used to discuss the participants’ decision-making regarding returning self-collected specimens for laboratory processing. Depending on the number and type of specimens returned by participants, the interviews were tailored to focus on factors that shaped their engagement or lack thereof.

### Qualitative Analysis

The in-depth interviews were transcribed using Scribie, checked for accuracy against the original audio files, and uploaded to Dedoose (Socio Cultural Research Consultants), a web-based platform for collaborative qualitative data analysis. Transcripts were analyzed using template analysis, a style of thematic analysis that involves developing an initial coding template using a subset of the data, applying it to further data, and refining it using an iterative process [54]. In this method, it is permissible to start with some a priori themes that are likely to be relevant to the analysis. First, an initial coding template that included a mix of themes identified in advance (eg, characteristics of the specimen self-collection box and additional information desired during the study) and themes identified from 3 interviews (one each from participants who returned all, some, and none of the specimens) was developed. Next, this template was applied to more transcripts, discussed among the research team, and iteratively revised based on the identification of newly emergent themes. Six overarching themes were coded: (1) influence of university branding on study credibility, (2) matters related to the transit and receipt of the specimen self-collection box, (3) internal attributes of the specimen self-collection box, (4) desire for instructional videos and additional test-related information, (5) preference-based tailoring of the specimen self-collection box, and (6) experiences with communications from the research team. Each theme also had subthemes that emerged from the participants’ narratives.

### Ethics Approval

The study protocol was reviewed and approved by the Institutional Review Board of the University of Michigan in Ann Arbor (HUM00153673). Electronic informed consent was obtained from all individual participants included in the study. Verbal consent for audio recording and transcription was also obtained from all individual participants at the beginning of each in-depth interview. Participants received US $40 for completing a web-based survey in the first phase of the study and US $40 for completing an in-depth interview in the second phase of the study.

## Results

### Sample Characteristics

The age of the participants of in-depth interviews ranged from 20 to 32 years, with the mean and median being 26 (SD 3.49) years. The sample of 25 participants was diverse with respect to race and ethnicity: 5 (20%) participants identified as non-Hispanic White; 4 (16%) as non-Hispanic Black; 7 (28%) identified as Asian; and 9 (36%) identified as Hispanic (irrespective of their race). Most participants had a college degree or a higher educational level (18/25, 72%), identified as gay (21/25, 84%), and were single (22/25, 88%). Regarding their recent sex behaviors, 40% (10/25) of participants engaged in condomless anal sex, and 76% (19/25) of participants engaged in condomless oral sex with ≥2 men in the past 3 months. Most participants (24/25, 96%) reported testing for HIV and two-thirds (17/25, 68%) reported testing for bacterial STIs in the past year. Finally, 44% (11/25) of participants indicated that they were using PrEP at the time of the study. This manuscript includes 30 verbatim excerpts from 18 participants (1-3 excerpts per participant).

### Influence of University Branding on Study Credibility

University branding was used throughout the study, including the social media advertisements, the web-based survey, and the specimen self-collection box. Our use of the well-recognized University of Michigan Block “M” logo instilled a sense of trust in the participants whose sentiments are evident in the following excerpts:

I think it was an ad, it was on Grindr, and it was just an ad like, “We’ll give you a gift card if you join our study,” and I’m kind of like, “Sounds a little sketchy,” but then I saw it was at University of Michigan, right? I think the university affiliation just makes me feel like a little more confident with it. I have an undergraduate degree. I’m currently going to graduate school. I’m comfortable with the university setting.

CAB161, aged 24 years, non-Hispanic White, returned some

I see University of Michigan, and I think, ‘Well, there you go. Respected institution.’ I think really pretty much any university that’s not like a private Christian college that was saying that we’re doing a study on this or we’re offering free sexual testing as part of a study I would trust them.

CAB002, aged 23 years, non-Hispanic White, returned none

I didn’t see any reason why a university would attempt to do anything out of the ordinary with them given HIPPA and the...What’s it called? When you have to do research and you have to go through the board for them to certify what you’re doing? I forget what that’s called, but I assume you wouldn’t be doing anything out of the ordinary because of that process.

CAB069, aged 27 years, Hispanic multiracial, returned some

The institutional review board–approved informed consent form and written instructions indicated that returned specimens would be processed at laboratories in Emory University and the University of California, San Francisco. Several participants noted that this information increased their confidence in the test results:

I would assume they would have integrity to give me the correct test results and everything.

CAB008, aged 28 years, non-Hispanic Black, returned all

I knew that there’s a university behind the study, and so I guess that kind of made me feel like the test was going back to a reputable source, to someone that was trustworthy, because it was for research purposes.

CAB012, aged 29 years, non-Hispanic Black, returned all

I thought it was reliable ’cause I thought that it was being sent to Emory and plus the study happened at U of M.

CAB086, aged 23 years, Hispanic, returned none

### Matters Related to the Transit and Receipt of the Specimen Self-collection Box

During the design phase, our research team recognized the importance of discretion while shipping the specimen self-collection box to prevent the inadvertent disclosure of the recipient’s involvement in a study on sexual health for GBMSM. Receiving a plain unmarked package with no reference to the nature of the contents was appreciated by several participants:

I did appreciate that because I do live with roommates, so I kind of prefer the discreetness to that. I did appreciate the fact that it was not labeled, and it was discreet and anonymous. So, yeah, I think that was pretty clever... But our neighbor saw it. I know other people saw the box, so it’s like... It was still, again, even though I was the one who picked it up first, I’m sure it was seen by others. So, the discreetness to it was still important.

CAB082, aged 32 years, Hispanic American Indian or Alaska Native, returned all

That definitely helped with anonymity, as I don’t live by myself. I don’t like people knowing what I get shipped to me, personally.

CAB122, aged 23 years, Asian, returned none

I think that was really great because it was able to just appear like any other package, so if people didn’t want the person delivering the mail or other people in the household to know what they were doing, they were able to have that privacy.

CAB120, aged 26 years, non-Hispanic White, returned none

One participant also appreciated the option of being able to provide a shipping address that differed from their residential address for logistical reasons:

I got it sent to my mother’s house. Because I get all my mail at my mother’s house instead of having it shipped to my place... Because sometimes if it’s a box, they’ll leave it on the top of the little mailbox thing because it’s like a little small slot for the mail at my building.

CAB091, aged 32 years, non-Hispanic Black, returned all

### Internal Attributes of the Specimen Self-collection Box

Several participants mentioned that upon opening the specimen self-collection box, they found its contents to be neatly organized:

It was well-organized, as far as everything was well-labeled and easy to follow.

CAB105, aged 29 years, Hispanic American Indian or Alaska Native, returned all

It looks like a very, very well-done prototype to me. I think something more, something that’s launched on a larger scale, you might be able to afford to do some more customized packaging inside of it that means there’s less empty space in it. But apart from that, it was really good, it looked very professionally done.

CAB002, aged 23 years, non-Hispanic White, returned none

Others commented that they appreciated our use of different colored bags with matching color-coded instructions to self-collect each type of specimen, as this helped them readily differentiate between the components inside the specimen self-collection box:

I like how it was separated, like each test was separated, and I believe it was color-coded. Like in the bag, that was also cool ’cause it allowed me to easily pin-point which test was for which direction and all that without having to do too much thinking or figuring out on my own.

CAB069, aged 27 years, Hispanic multiracial, returned some

I liked the colors. It kinda reminded me of Google...It was good. I never had any missing items, everything was pretty clear. The instructions, the layout...You open the box, you have everything in there, you go through the tests one by one. Yeah, so everything was pretty clear.

CAB044, aged 28 years, Hispanic multiracial, returned all

### Desire for Instructional Videos and Additional Test-Related Information

When asked how our specimen self-collection instructions could be enhanced, some participants recommended the inclusion of prerecorded instructional videos in addition to written instructions, especially for the finger-stick blood sample:

I think having video instructions where you can actually do the test with someone, that you can just do it while they do it on the video, that’ll be nice... Then you kinda see that in real action versus having to comprehend what’s on paper.

CAB014, aged 27 years, Asian, returned none

A video for the blood sample, just to ease anxiety and show people that it’s not what you think it is, like in a doctor’s office, would be helpful. But for something like the rectal swab, a printed thing with a couple of pictures of how far to stick it up your... you know, how far to stick it up is just fine, and you don’t really need a video for that. And let’s say for like the throat, printed text... I think that would be sufficient. But for a blood sample, since that’s a little more invasive, maybe a video would help.

CAB086, aged 23 years, Hispanic, returned none

Others discussed the need to highlight the importance of triple-site bacterial STI testing:

I think that information (on triple-site bacterial STI testing) would have helped me. I don’t know if it would motivate me per se, but I think it would help me understand... “Okay, now I’m doing this for the following purposes” and not so much as “Oh, if I do this and this but that’s retesting the same thing. Why am I doing both?” But if I knew if different things were used for different STIs, then in some ways, I guess I would feel like “Okay, there is a reason for me to be doing all these different tests so that I know my status around all those different STIs.

CAB014, aged 27 years, Asian, returned none

Several participants did not remember why they were being asked to provide a hair sample by the time they received the specimen self-collection box, suggesting that the inclusion of a reminder on the types of testing that would and would not be performed on hair samples might be helpful:

I honestly don’t know what you can get from hair samples other than previous drug testing, so I didn’t know what information you could actually glean from hair sample. But I figured if you asked for it, there must be something.

CAB048, aged 22 years, non-Hispanic White, returned some

That one’s different. I didn’t understand that one. I never really did get any samples. The only thing I could think about that one, was more like DNA... I was kind of skeptical of it.

CAB033, aged 27 years, non-Hispanic Black, returned none

I did think like, Okay, why are you actually needing to collect hair if it’s an HIV study, or if it’s being marketed as an at-home HIV test?

CAB024, aged 26 years, non-Hispanic White, returned none

### Preference-Based Tailoring of the Specimen Self-collection Box

One theme that emerged from the participants’ narratives was their desire for a customized specimen self-collection box that only included instructions and materials for those tests that they were interested in completing at that time:

I would definitely prefer to do just the four and leave out the blood... Not that I’m opposed to doing it at all, I would just prefer to do it with less frequency than the others.

CAB082, aged 32 years, Hispanic American Indian or Alaska Native, returned all

In addition to enhancing the overall experience, a potential benefit of tailoring the specimen self-collection box could be an improved rate of specimen return. Some participants described how they felt overwhelmed at the thought of self-collecting 5 specimens, which prevented them from proceeding any further:

I was expecting something smaller, so it was kind of overwhelming and it probably, it kind of influenced my decision about whether to continue on with it or not... Because I thought I had to send all of them together, so I just decided not to send any of it.

CAB075, aged 23 years, Hispanic, returned none

I probably wrongly assumed that I should send in all five, and that they were mandatory, but taking two seconds to think about it, I know that it... Yeah, it didn’t really matter, or I could have done some, not all.

CAB024, aged 26 years, non-Hispanic White, returned none

Participants who were using PrEP also discussed that their established routine of clinic-based HIV testing reduced their motivation to undertake home-based HIV testing:

Because of PrEP, I’m less inclined to seek out DIY HIV testing at this point...I take PrEP, so I have to go in for quarterly check-ups anyway for that. I had got one (HIV test) done recently when the box arrived and I thought, “I’ll wait until a couple of weeks have passed, so that this is actually useful.”

CAB002, aged 23 years, non-Hispanic White, returned none

I get tested for HIV regularly, and I’m on PrEP so I wasn’t dying to know if I had HIV. Does that make sense? Maybe if I didn’t know, I would be much more inclined to go through the pricking. But since I know, I didn’t have the curiosity.

CAB051, aged 30 years, Hispanic multiracial, returned some

One participant recommended contacting the participants before shipping them a specimen self-collection box to inquire about which of the 5 tests they desired:

I would send them [participants] an email, and then depending on what results, what they send back, I would send them the tests that they wanted to do.

CAB044, aged 28 years, Hispanic multiracial, returned all

### Experiences With Communications From the Research Team

During discussions regarding their experiences with receiving communications from the research team at different time points, participants offered suggestions on types of further support that could help improve study engagement.

One participant recommended the addition of real-time videoconferencing at the beginning of the study to introduce the research team (instead of potentially including a prerecorded welcome video), as that would allow new participants to ask questions or discuss concerns:

If I’m concerned about something I could ask you on the spot, which is different than if you guys just sent me a video. So, it would have made me more comfortable speaking to someone, ’cause if I have a question or something, ask them, where the video doesn’t... Really wouldn’t answer any of those.

CAB075, aged 23 years, Hispanic, returned none

Some participants discussed how receiving personalized reminders via their preferred modes of communication (eg, texts and emails) following the receipt of the specimen self-collection box might have increased their likelihood of returning specimens:

I would say text reminders would have been fine, maybe emails depending on people’s preferences for contact, and just trying to make sure that regardless of if they were automatic messaging, making it seem as though it’s actually a person would be important so it’s not just a bot.

CAB024, aged 26 years, non-Hispanic White, returned none

I think a reminder would have definitely been helpful in terms of just bringing it back in my mind again ‘Oh, I got this thing in my room that I need to do.’ But yes, I mean... I don’t know how it’s actually actualized in a real world setting versus a research setting, where if someone just buys it and no one is reminding them to do it.

CAB014, aged 27 years, Asian, returned none

Receiving their test results via multiple communication channels was also valued by participants, as it provided an opportunity to ask questions over the phone immediately and to access a copy of their test results via email later:

Okay, I really liked that you emailed the test results. I loved that ’cause you can have it with you and if you have to go get treatment or something, you could just pull it up on your phone. So, I liked that... I would actually prefer both (phone call and email), but just have an email as a backup... Just so I can hear somebody say it and then if I have a question or something I could immediately ask the question.

CAB008, aged 28 years, non-Hispanic Black, returned all

## Discussion

### Principal Findings

Several aspects of the implementation of Project Caboodle! were appreciated by the participants, as evidenced by their positive feedback during the in-depth interviews. Using the University of Michigan Block “M” logo in all study materials enhanced credibility; shipping the specimen self-collection box in plain unmarked packaging promoted discretion; and using different colored bags with matching color-coded instructions to self-collect each type of specimen minimized the potential for confusion. Participants also offered some constructive commentary, providing important lessons for future refinements to our processes that could help improve the rates of self-collected specimen return in subsequent work with GBMSM. They recommended including prerecorded instructional videos to supplement the written instructions, providing information on the importance of triple-site bacterial STI testing, and adding a reminder of the types of testing that would and would not be conducted on hair samples. They also suggested tailoring the specimen self-collection box based on individual preferences, adding real-time videoconferencing at the beginning of the study to introduce the research team, and sending personalized reminders following the delivery of the specimen self-collection box as practical strategies to bolster the likelihood of specimen return.

One feature of our study that contributed to its success was our consistent use of university branding on the social media advertisements, the web-based survey, and the specimen self-collection box. University academic logos are unique visual representations that signal institutional identity and can instill a sense of trust [55] and perceived competence [56] in the general public. Participants clearly valued the information regarding who was behind the research from the time point of their initial contact with one of our study’s advertisements on Facebook or Grindr all the way through to their return of self-collected specimens to university-based laboratories and the receipt of test results. We were successful in recruiting a racially and ethnically diverse sample of 100 GBMSM from across the United States via social media advertising, and the proportion of specimens returned for laboratory processing in our study (51/100, 51%) was higher than that in 6 other studies (30%) recently completed with GBMSM that did not incentivize specimen return either [57]. Including university or institutional branding at different stages of participant interaction is an effective way to enhance a study’s credibility and may facilitate the recruitment and retention of participants in future web-based HIV and bacterial STI prevention research.

The participants in our study also appreciated receiving the specimen self-collection box at their residence in plain unmarked packaging. This afforded privacy and prevented an inadvertent disclosure of their involvement in sexual health research to the mail carrier or to someone they lived with such as a roommate or parent. Given the stigma associated with HIV and bacterial STI testing [58,59], as well as PrEP use [60,61] among GBMSM, the use of discreet packaging reduces the potential for discomfort or harm if the contents of the package are revealed to someone other than the participant. For extra discretion, neither our primary package (ie, the specimen self-collection box containing instructions and materials) nor our secondary package (ie, the cardboard United Parcel Service box used for shipping the specimen self-collection box) made any reference to the nature of the contents. Studies with multiple self-collection points would particularly benefit from applying discretion while shipping packages to maintain participant’s confidentiality and possibly reduce attrition. It might also help to offer participants the option of providing an alternative shipping address, such as a Post Office box or self-service parcel locker in case they have concerns about receiving a shipment at their residential address.

Internal attributes of the specimen self-collection box that were well received by participants included its neat organization and our use of different colored bags with matching color-coded instructions for different types of specimens: red for a finger-stick blood sample, blue for a pharyngeal swab, green for a rectal swab, yellow for a urine specimen, and black for a hair sample. Of note, the bags and instructions were purposefully color-coded to align with the Project Caboodle! study logo [52]. Color coding is an effective strategy to enable recipients to easily distinguish between separate components included in a package [62,63] and could help reduce ambiguity during the process of self-collecting different types of specimens. Our research team strove for a minimalistic package design while ensuring the harmoniousness of various components inside the specimen self-collection box [64]. It is important that the contents of the package be well organized and easy to navigate independently, particularly in large-scale studies or public health programs in which there might be minimal interaction between the researchers or practitioners and participants or clients, respectively.

Shifting focus to avenues for improvement, some participants recommended the inclusion of prerecorded instructional videos in addition to written instructions, especially for the finger-stick blood sample, to allay their anxiety (by demonstrating how finger-stick blood self-collection differs from phlebotomy conducted in a clinic) and bolster their self-efficacy (by offering an opportunity to perform self-collection alongside someone in the video). We acknowledge that providing access to prerecorded instructional videos is becoming increasingly common in specimen self-collection research [65-67] and concur with those who emphasize that supplemental instructional resources should be accurate and easy to comprehend (including their availability in multiple languages) and should provide clear guidance on how to handle specimens after self-collection [23,68]. On the basis of participants’ feedback, it might also be beneficial to highlight the importance of triple-site bacterial STI testing and reiterate the types of testing that would and would not be performed on hair samples. Extragenital gonococcal and chlamydial infections are prevalent among GBMSM [9-11] and may remain undiagnosed if urine-only screening is performed [12,13]. Educating sexually active GBMSM about the importance of triple-site bacterial STI testing might help improve return rates of pharyngeal swabs, rectal swabs, and urine specimens in subsequent studies. Similarly, reminding participants that returned hair samples would only be used for PrEP drug–level testing (and not for substance use or DNA testing) could alleviate possible skepticism and resistance to returning this specimen.

Another recommendation put forth by participants was potentially customizing the specimen self-collection box to include only instructions and materials for those tests that they were interested in completing at that time. The prospect of self-collecting 5 specimens was reported to be overwhelming by some participants, and it negatively influenced the likelihood of return despite having a choice to return only those specimens that they felt comfortable returning. Similar results were noted in another recent study with young GBMSM who were offered the option to self-collect and return a finger-stick blood sample for syphilis testing along with pharyngeal, urethral, and rectal swabs for gonorrhea and chlamydia testing [66]. Despite intending to return specimens, a subset of participants kept postponing self-collection, as they were overwhelmed by the process. Some participants in our study were also apprehensive about pricking their own finger. Those who were using PrEP were less inclined to return a finger-stick blood sample for HIV testing because of their established routine of testing at a clinic every 3 months while refilling their prescription. Contacting participants before shipping them a specimen self-collection box to inquire regarding which tests they desired to be performed might help improve return rates and reduce wastage of material, financial, and personnel resources.

Participants in our study also offered recommendations on the addition of communication strategies that could help improve engagement at multiple time points. Connecting with the research team via real-time videoconferencing at the beginning of the study would provide new participants with an opportunity to have their questions or concerns addressed and set the foundation for developing a rapport. Longitudinal studies with GBMSM should consider incorporating onboarding sessions conducted via real-time video conferencing if resources permit. Participants who did not return any specimens mentioned that receiving personalized reminders via their preferred modes of communication (eg, texts and emails) following the receipt of the specimen self-collection box might have prompted them to return the specimens. The reason we did not include reminders (or incentives) was to avoid influencing return rates in our acceptability and feasibility study. However, reminders have been shown to influence behaviors across a spectrum of health care issues and can help promote study engagement [69,70]. Returning test results via multiple communication channels is also a good practice as a phone call or real-time videoconferencing allows for the immediate provision of support and resources (especially in the case of a positive result) and a text or email allows for access to a copy of the results in the future.

### Study Limitations

Caution should be exercised in generalizing our findings because our recruitment was restricted to GBMSM who were aged between 18 and 34 years and had accounts on Facebook or Grindr. Their opinions on factors shaping study engagement or lack thereof may differ from users of other social media or dating platforms and from those who do not have a presence on the web. In addition, only 3% (3/100) of the participants in our study resided in areas designated as rural by the Federal Office of Rural Health Policy, and none of them were interviewed. This limitation precludes our understanding of issues unique to rural residents (eg, difficulty returning specimens by mail in case they have to travel long distances to a shipping facility and paying a surcharge for package pickup if they live in an area deemed to be less accessible by shipping carriers). Our interviews were conducted only in English, which could have posed a language barrier for some participants. Finally, most interviews were completed before the COVID-19–related stay-at-home mandates came into effect, and it is possible that additional themes and subthemes may have emerged had we interacted with the participants during the pandemic.

### Conclusions

Soliciting self-collected specimens for HIV, triple-site gonorrhea, and chlamydia, and PrEP drug–level testing from GBMSM might hold promise as a remote monitoring strategy for individuals at elevated risk. In-depth interviews with a subset of participants who returned all, some, or none of the specimens in our exploratory study offered valuable insights into aspects that facilitated their engagement as well as areas for potential improvement. Our results have pragmatic implications for the design of subsequent large-scale studies and public health programs for home-based HIV, bacterial STI, and PrEP adherence testing. This comprehensive overview of the key lessons learned from our study’s implementation and recommendations offered by participants could help maximize the rates of self-collected specimen return in future web-based HIV and bacterial STI prevention research and practice.

## Abbreviations

GBMSM: gay, bisexual, and other men who have sex with men
PrEP: preexposure prophylaxis
STI: sexually transmitted infection

## Data Availability

The data sets generated or analyzed during this study are available from the corresponding author on reasonable request.
